# Supplementary material for: Nicheformer: a foundation model for single-cell and spatial omics
Source: Nat Methods. 2025 Oct 30;22(12):2525–38. doi: 10.1038/s41592-025-02814-z (PMC12695652; doi:10.1038/s41592-025-02814-z)
Supplement: Supplementary file 1 — Supplementary Notes 1 and 2 and Supplementary Tables 1–5 [file 41592_2025_2814_MOESM1_ESM.pdf]

nature methods

Supplementary information

https://doi.org/10.1038/s41592-025-02814-z

Nicheformer: a foundation model for single-cell and spatial omics

In the format provided by the authors and unedited

Supplementary information

Supplementary Notes

Supplementary Note 1 | Impact of dataset size, compute-per-sample, and scaling on Nicheformer performance

To better understand the factors contributing to model performance, we trained Nicheformer models on subsets of the SpatialCorpus-110M dataset with different sizes (1% and 3%) but fixed total compute budget. Surprisingly, models trained with only ~1% of the data exhibited no notable decrease in performance relative to models trained on the full dataset. Moreover, the model trained on the smaller 1% subset slightly outperformed the one trained on 3%.

We attribute this to differences in compute-per-sample: with the same total compute, the smaller dataset allowed for more optimization steps per sample, leading to better learning. In contrast, the larger subset, while containing more data, diluted the compute effort across more samples, resulting in slightly less refined representations.

These findings indicate that computational efficiency per sample—not just dataset size—is a critical factor for effective training. This supports the view that, without appropriately scaling compute with dataset size, large models may remain undertrained on certain subsets, such as liver cells in SpatialCorpus-110M.

This observation aligns with scaling laws established for large language models, where model performance is jointly governed by model size, dataset size, and compute budget. In line with these theories, our results suggest that a modest amount of focused pretraining on underrepresented tissues can effectively compensate for undertraining due to limited initial compute allocation.

Supplementary Note 2 | Detailed analysis of spatial-to-dissociated transfer in motor cortex

We evaluated Nicheformer's ability to transfer spatially-defined cell type labels from the MERFISH mouse brain dataset onto dissociated scRNA-seq profiles from primary motor cortex cells. Among the 33 MERFISH cell types, Nicheformer accurately identified the nine motor cortex–related types relevant for the scRNA-seq dataset (Fig. 4E, Suppl. Fig. 8I). Classification uncertainty, calculated from the full output distribution (Methods), was generally low, indicating high agreement between predicted and original annotations (Fig. 4E, I).

Non-neuronal cells were consistently mapped to their correct spatial identities. Glutamatergic (Glut) neurons, the most abundant cell type (Fig. 4H), were also correctly matched overall. However, for some Glut subtypes corresponding to deeper cortical layers (e.g., L6b, L6 CT, L5/6 NP), misclassification into midbrain glutamatergic (MB Glut) subtypes occurred (Suppl. Fig. 8I). This likely reflects underlying transcriptional heterogeneity in the MERFISH training data: MB Glut encompasses 657 subclusters versus only 83 for NP-CT-L6b Glut, despite similar or larger absolute cell counts (MB Glut: 88,169 cells; NP-CT-L6b Glut: 174,616 cells).

For niche label prediction, Nicheformer correctly assigned all expected labels for non-neuronal cells with low uncertainty (Fig. 4F, J, Suppl. Fig. 8J). Neurons were predominantly assigned to either the pallium glutamatergic or subpallium GABAergic niches, although higher uncertainty was observed for excitatory neurons. Some misclassification is likely due to the spatial proximity and overlap between the pallium and subpallium regions (Suppl. Fig. 8L-M).

For regional prediction, Nicheformer correctly predicted the isocortex as the primary region for the majority of cells (Fig. 4G, K, Suppl. Fig. 8K). Minor spillover into adjacent regions such as cortical subplate (CTXsp), olfactory area (OLF), and white matter was observed. These could plausibly result from tissue dissection artifacts, where neighboring regions are inadvertently captured. Notably, prediction accuracy for non-neuronal cells was slightly lower, possibly reflecting their reduced transcriptional regional specificity compared to neurons.

Supplementary Tables

**Hyperparameter**	**Value**
Optimizer	[**AdamW**, Adam]
Optimizer momentum	$\beta_1 = 0.9$,<br>$\beta_2 = 0.99$
Maximum learning rate	**1e-3**
Minimum learning rate	[**1e-5**, 0]
Weight decay	[**0.1**, 0.01, 0.0]
Dropout	[0.15, 0.10, **0.0**]]
Maximum gradient clipping	[**1.0**, 0.7, 0.5, 0.0]
Minimum gradient clipping	[**0.5**, 0.0]
Warmup iterations	**100K**
Batch size	**9**
Gradient accumulation	[**10**, 0]
Precision	[**bfloat16**, float32]
Embedding dimensionality	[**512**, 256]
FFN dimensionality	[**1024**, 512]
Transformer blocks	[**12**, 8, 6]

Attention heads	[**16**, 12, 8]
Gene tokens	**20310**
Contextual tokens	**15**
Number of parameters	[**49.3**, 40.9, 15.1] M

Suppl. Table 1 | Overview of pretraining architecture hyperparameters used for the final Nicheformer pretraining model. Shown are the model hyperparameters screened for the Nicheformer pretraining with parameters highlighted in bold being the final parameter selection.

	Nicheformer	Geneformer	CellPLM
**Dataset size**	SpatialCorpus-110M (110 million cells)	Genecorpus (30 million cells)	11 million cells
**Tissue, species**	Cross-tissue, humans & mouse	Cross-tissue, human	Cross-tissue, human
**Modalities**	Dissociated & Spatial	Dissociated	Dissociated & Spatial
**Tokenization**	Gene rank-based value encoding	Gene rank-based value encoding	Cell tokenization
**Vocabulary size**	20,310 protein-coding genes, (16,981 orthologous genes, 3,178 human-specific and 151 mouse-specific)	25,424 protein-coding genes	13, 500 common gene set
**Context length**	1,500	2,048	Uses a batch of cells as context
**Transformer units**	12	6	4
**Embedding dimension**	512	256	512
**Total parameters**	~ 50 million	~ 10 million	~ 82 million
**Dissociated downstream tasks**	Cell type annotation	Gene function prediction, cell type annotation, GRN inference	Cell type annotation, genetic perturbation effect prediction
**Spatial downstream tasks**	Niche and region label prediction, neighborhood composition prediction, cellular density prediction	Not considered	Gene expression imputation

Suppl. Table 2 | Comparison of Nicheformer architecture to Geneformer and CellPLM. Shown are the pretraining dataset specifications, model specifications and evaluated downstream tasks for Nicheformer compared to Geneformer and CellPLM.

GEO ID	DOI	Number of cells	Tissue	Assay	Author
**GSE117824_human**	10.1038/s41586-019-1367-0	51937	blood; bone marrow	10x 3' v2	Nam_2019
**GSE117824_mouse**	10.1038/s41586-019-1367-0	738	hematopoietic cell	10x 3' v2	Nam_2019
**GSE118068**	10.1038/s41586-019-1158-7	62893	brain	10x 3' v2	Vladoiu_2019
**GSE119940**	10.1038/s41590-019-0403-4	46856	spleen; bone marrow	10x 3' v2	Yao_2019
**GSE124952**	10.1038/s41467-019-12054-3	43795	brain	10x transcription profiling	Bhattacherjee_2019
**GSE126060**	10.1038/s41467-019-14172-4	41701	tendon	10x 3' v2	Sorkin_2020
**GSE128423**	10.1016/j.cell.2019.04.040	135004	bone marrow; bone tissue	10x 3' v2	Baryawno_2019
**GSE128761**	10.1016/j.stem.2020.08.001	231485	liver; bone marrow	10x 3' v2	Li_2020
**GSE128987**	10.1038/s41556-020-00619-0	25932	female reproductive system	10x 3' v2	Chumduri_2021
**GSE129826**	10.1016/j.celrep.2019.10.073	106670	lymphoblast	10x 3' v2	Xie_2019
**GSE130593**	10.1242/dev.183251	53927	testis	10x 3' v2	Tan_2020
**GSE130822**	10.1016/j.stem.2019.12.011	5401	intestine	10x 3' v3	Murata_2020
**GSE130879**	10.1016/j.immuni.2019.12.002	31614	lymph node; blood; adipose tissue; lung; skin of body; spleen; liver; bone marrow	10x 3' v2	Delacher_2020
**GSE130888**	10.1126/scitranslmed.aav5341	96446	peritoneum	10x 3' transcription profiling	Si_2019
**GSE131339**	10.1038/s41467-019-13465-y	42213	thymus	10x 3' v2	Cowan_2019
**GSE131996**	10.1016/j.immuni.2019.06.009	32805	lung	10x 3' v2	Nagashima_2019
**GSE132355**	10.1038/s41467-020-18231-z	341322	hypothalamus	10x transcription profiling	Kim_2020
**GSE133531**	10.1038/s41588-019-0531-7	65589	brain	10x 3' v2	Jessa_2019
**GSE134571**	10.1038/s41586-019-1535-2	13661	embryonic stem cell; amniotic stem cell	10x 3' v2	Zheng_2019
**GSE135310**	10.1161/CIRCRESAHA.120.317200	22630	heart	10x 3' v2; 10x 3' v3	Vafadarnejad_2020
**GSE135326**	10.1038/s41586-0	280410	brain	10x 3' v3	Chu_2019

	19-1644-y				
**GSE135356**	10.1038/s41467-020-18957-w	60841	endothelial cell of umbilical vein	10x 3' v2	Calandrelli_2020
**GSE135414**	10.1016/j.celrep.2020.01.075	10509	retina	10x 3' v2	Jorstad_2020
**GSE136394**	10.1158/2326-6066.CIR-19-0299	73585	intestine; blood	10x 5' transcription profiling	Lu_2019
**GSE136441**	10.1073/pnas.2005570117	52542	gonad; ovary	10x 3' v2	Niu_2020
**GSE137026**	10.1084/jem.20220126	43363	lung	10x 3' v2	Liu_2022
**GSE139168**	10.1126/sciadv.aba9950	28731	heart	10x 3' v3	Hatzistergos_2020
**GSE140510**	10.1038/s41591-019-0695-9	90647	brain	10x 5' transcription profiling	Zhou_2020
**GSE140628**	10.1158/2159-8290.CD-19-0958	14563	pancreas	10x transcription profiling	Zhang_2020
**GSE141471**	10.1038/s41467-021-27899-w	54855	hindlimb	10x 3' v3	Dutrow_2022
**GSE141526**	10.1126/sciadv.abm7981	56162	skin of body	10x 3' v2	Yao_2020
**GSE141552**	10.1093/hmg/ddaa038	53616	brain	10x 3' v2	Brenner_2020
**GSE141784**	10.1084/jem.20192362	106681	pancreas	10x 3' v2	Zakharov_2020
**GSE142143**	10.1038/s41419-022-04693-0	30659	brain	10x 3' v2	Lin_2022
**GSE142797**	10.1101/2020.04.27.063503	137873	brain	10x 3' v3	Winkel_2020
**GSE143293**	10.1038/s41586-020-3017-y	19345	bone marrow	10x transcription profiling	Yusufova_2020
**GSE145216**	10.1016/j.cell.2020.03.004	98191	vagus nerve	10x 3' v3	Prescott_2020
**GSE145251**	10.1016/j.stem.2022.03.001	91242	motor neuron; endodermal cell; cardiac muscle cell	10x 3' v3	Kong_2022
**GSE145326**	10.1172/JCI130323	63888	breast	10x 3' v3	Garcia-Recio_2020
**GSE145689**	10.1681/ASN.2020070930	324635	kidney	10x 3' v2	Hinze_2021
**GSE145866**	10.1016/j.celrep.2020.107952	420146	intestine	10x 3' v2	Sheng_2020
**GSE146122**	10.1016/j.cell.2020.03.015	22194	skin epidermis	10x 3' v2	Dekoninck_2020
**GSE146138**	10.1053/j.gastro.2020.09.011	110107	intestine	10x 3' v2	Lähde_2021
**GSE146194**	10.1016/j.cell.2022.05.013	669018	blood	10x 3' v2	Replogle_2020

**GSE146298**	10.1016/j.celrep.2020.03.059	159906	brain	10x 3' v2	Darbandi_2020
**GSE146512**	10.1016/j.celrep.2020.108027	100639	ovarian follicle	10x 3' v3	Man_2020
**GSE148339**	10.1016/j.jcmgh.2020.07.012	16714	liver	10x 3' v3	Nault_2021
**GSE148978**	10.1016/j.immuni.2020.10.024	135297	thymus	10x 5' transcription profiling	Chopp_2020
**GSE149040**	10.1038/s41467-021-21704-4	86719	retina	10x 3' v2	Wu_F_2021
**GSE149201**	10.1158/2159-8290.CD-20-0461	90263	bone tissue	10x 3' v3	Khazaei_2020
**GSE149356**	10.1126/sciimmunol.abf0125	28775	blood	10x 5' transcription profiling	Tan_2021
**GSE149931**	10.1038/s41587-020-00763-w	55202	brain	10x 3' v3	Miura_2020
**GSE150708**	10.21203/rs.3.rs-62758/v1	32701	lung	10x 3' v2	Duan_2020
**GSE150871**	10.1038/s41586-020-2795-6	127783	spinal cord	10x 3' v3	Li_Y_2020
**GSE150995**	10.1172/JCI136142	13983	tendon	10x 3' v3	Huber_2020
**GSE151186**	10.1016/j.stem.2020.10.003	5256	embryonic stem cell	10x 3' v3	Mikryukov_2021
**GSE152325**	10.1038/s41556-020-00617-2	297808	intestine	10x 3' v2	Böttcher_2021
**GSE152573**	10.1182/blood.2020007747	27176	bone marrow	10x 3' v2	Zheng_2020
**GSE152988**	10.1038/s41593-021-00862-0	96637	neuron	10x 3' v3	Tian_2021
**GSE152999**	10.1038/s41467-020-20351-5	174462	intestine	10x transcription profiling	Sarvestani_2021
**GSE153099**	10.1016/j.stemcr.2020.12.018	43143	retina	10x 3' v2	Kruczek_2021
**GSE153117**	10.1038/s41467-021-26069-2	24057	thymus	10x 3' v2	Cordero_2021
**GSE153274**	10.1038/s41467-021-22021-6	42996	spermatogonium	10x 3' v3	Zhang_2021
**GSE153288**	10.1038/s41467-020-17544-3	39848	thymus	10x 3' transcription profiling	Lebel_2020
**GSE153762**	10.7554/eLife.60223	17384	sciatic nerve	10x 3' v3	Kalinski_2020
**GSE153770**	10.1016/j.celrep.2020.108004	32412	embryo; liver	10x 3' v2; 10x 3' v3	Simic_2020
**GSE153802**		254501	intestine	10x 3' v3	Hong_2020
**GSE154196**	10.15252/embj.2020106423	14296	brain	10x 3' transcription profiling	Jönsson_2021

**GSE154359**	10.1016/j.stem.2020.08.015	24845	skin fibroblast	10x 3' v2	Cates_2021
**GSE154386**	10.1371/journal.ppat.1009240	200192	blood	10x 5' v2	Waickman_2021
**GSE154567**	10.1016/j.celrep.2020.108590	85064	blood	10x 3' v3	Yao_2021
**GSE154579**	10.1038/s41467-020-19234-6	97363	skin of body	10x 3' v2; 10x 3' transcription profiling	Lin_2020
**GSE154932**	10.1088/1478-3975/abb09c	14964	mammary gland epithelial cell	10x 3' v2	Johnson_2020
**GSE155226**	10.1126/scitranslmed.abf7872	47066	heart	10x 3' v3	Pérez-Bermejo_2020
**GSE155340**	10.1126/sciimmunol.abb5168	5167	bone marrow	10x transcription profiling	Aykut_2020
**GSE155788**	10.7554/eLife.61413	35453	brain	10x 3' transcription profiling	Orsenigo_2020
**GSE155850**	10.1172/jci.insight.139932	88559	skin of body	10x 3' v3	Lowe_2020
**GSE156136**	10.1016/j.cell.2020.09.062	38925	bone marrow	10x 3' transcription profiling	Khan_2020
**GSE156183**	10.1073/pnas.2017742118	94488	brain	10x 3' v2	Ellwanger_2021
**GSE156245**	10.1038/s41586-021-03283-y	23834	colon	10x 3' v2	Wu_2021
**GSE156285**	10.1126/sciimmunol.abc6259	36091	upper respiratory conduit	10x 3' transcription profiling	Hung_2020
**GSE156920**	10.1038/s41467-021-23320-8	12967	embryonic stem cell	10x 3' v2	Liu_2021
**GSE157244**	10.1161/JAHA.120.019019	153337	blood; heart; bone marrow	10x transcription profiling	Calcagno_2021
**GSE157292**	10.1172/jci.insight.141321	60181	kidney	10x transcription profiling	Dangi_2020
**GSE157362**	10.1242/dev.197111	3141	testis	10x 3' v3	Webster_2021
**GSE157525**	10.1101/gad.339978.120	37665	cerebral cortex	10x 3' v2	Parisian_2020
**GSE157771**	10.1038/s41593-020-00745-w	102376	brain; spleen	10x 3' v2	Jin_2021
**GSE157773**		48705	mesenchymal stem cell; embryonic stem cell; skeletal muscle satellite myogenic cell	10x 3' transcription profiling	Han_2020
**GSE157977**	10.1126/science.aaz6063	89737	brain	10x 3' v2	Jin_2020
**GSE158038**	10.1038/s41467-021-22210-3	96600	blood; lung	10x 5' transcription profiling	Ferreira-Gomes_2021

**GSE158192**	10.1038/s41467-021-22817-6	88389	lung	10x 3' v2	Little_2021
**GSE158356_mouse**	10.26508/lsa.202000935	98297	pancreas	10x transcription profiling	Kemp_2021
**GSE158450**	10.1038/s41467-020-20343-5	29375	brain	10x 3' v2	Joglekar_2021
**GSE159354**	10.1016/j.xcrm.2020.100140	184019	lung	10x 3' v2	Gao_2020
**GSE159519**	10.1016/j.cell.2020.10.030	71279	epithelial cell of lung	10x 5' v1	Daniloski_2021
**GSE159977**	10.1038/s41586-021-03362-0	112566	liver	10x 3' transcription profiling	Pfister_2021
**GSE160061**	10.1111/cpr.12933	11271	germ cell	10x 3' v3	Yang_2021
**GSE160097**	10.1002/eji.202048797	35164	blood; synovial fluid	10x 5' transcription profiling	Maschmeyer_2021
**GSE160098**	10.1038/s41467-023-38647-7	68404	myoblast	10x 3' v3	Sunadome_2023
**GSE160664**	10.1164/rccm.202008-3198OC	7368	lung	10x 3' v2	Okuda_2021
**GSE160729**	10.1016/j.cmet.2020.12.004	442800	adipose tissue	10x 3' v2	Sárvári_2021
**GSE160772**	10.1096/fj.202002123R	6379	endometrium	10x 3' v2	Kirkwood_2021
**GSE161066**	10.3389/fphys.2021.637924	27111	immature Schwann cell; amnion mesenchymal stem cell	10x 3' v2	Wei_2021
**GSE161227**	10.1084/jem.20212479	257991	brain	10x 3' v3	Zhao_2022
**GSE161230**		124799	brain	10x 3' v3	Zhao_2020
**GSE161363**	10.1126/science.abc1944	85227	epithelial cell of lung	10x 3' transcription profiling	Quinn_2021
**GSE161685**	10.1172/jci.insight.144294	20071	lung	10x 3' transcription profiling	Gally_2021
**GSE161937**	10.1073/pnas.1915389116	41512	uterus	10x 3' v3	Fitzgerald_2019
**GSE162073**	10.1084/jem.20200844	82138	breast	10x 3' v3	Xu_2021
**GSE162807_human**	10.1038/s41467-022-28473-8	4605	spinal cord	10x 5' transcription profiling	Tansley_2022
**GSE162807_mouse**	10.1038/s41467-022-28473-8	239635	spinal cord	10x 5' transcription profiling	Tansley_2022
**GSE163018**	10.1038/s41421-021-00266-1	49534	brain	10x 3' transcription profiling	Ziffra_2021

**GSE163278**	10.1172/jci.insight.127807	63765	bone marrow	10x transcription profiling	Bailur_2019
**GSE163650**	10.1371/journal.pone.0244743	39794	liver	10x 3' v2	Taylor_2021
**GSE163668**	10.1038/s41586-021-03234-7	189079	blood	10x 5' v1	Combes_2021
**GSE163701**	10.1038/s41698-021-00160-9	9128	adipose tissue	10x 3' v3	Su_2021
**GSE163830**		49527	adipose tissue	10x 3' v3	Gupta_2020
**GSE163919**		12685	lung	10x 5' v1	Banovich_2020
**GSE164044**	10.1016/j.neuron.2019.08.002	22559	retina	10x 3' v3	Norrie_2019
**GSE164573**	10.1038/s41467-021-22842-5	34439	skeletal muscle tissue	10x 3' v2	Julien_2021
**GSE165551**	10.7554/eLife.67436	54367	brain	10x 3' v2	Cebrian-Silla_2021
**GSE165554**	10.7554/eLife.67436	35025	brain	10x 3' v3	Cebrian-Silla_2021
**GSE166218**	10.1093/neuonc/noac138	161021	brain	10x 3' v2	Friedrich_2023
**GSE166262**	10.1038/s41588-021-00818-x	53214	embryo	10x 3' v3	Kameneva_2021
**GSE166525**	10.1186/s13046-023-02686-1	113518	brain	10x 3' v3	Liu_2023
**GSE166797**	10.1073/pnas.2023070118	7227	lymphoblast	10x transcription profiling	Wu_2021_2
**GSE166992**	10.1016/j.celrep.2021.108863	63895	blood	10x 5' transcription profiling	Thompson_2021
**GSE167595**	10.1158/1940-6207.CAPR-21-0378	62644	intestine	10x 5' transcription profiling	Yang_2022
**GSE167992**	10.1016/j.stem.2021.04.003	4787	cornea	10x 3' v2	Altshulerls_2021
**GSE168732**	10.1038/s41467-021-25771-5	106453	blood	10x 3' transcription profiling	Wang_2021
**GSE168758**	10.1016/j.jhep.2021.03.029	101050	liver	10x 3' v3	Reich_2021
**GSE169718**	10.1016/j.devcel.2021.12.012	51289	intestine	10x 3' v3	Ohara_2022
**GSE172127**	10.1038/s41421-021-00266-1	18061	liver	10x 3' v2	Lu_2021
**GSE200218**	10.1016/j.cell.2022.06.007	191032	brain; axillary lymph node; subcutaneous adipose tissue	10x 5' transcription profiling	Biermann_2022
**GSE225278**	10.1038/s41467-023-38704-1	201525	pluripotent stem cell	10x 3' v3	Neavin_2023
**GSE114687**	10.1038/s41588-019-0489-5	75735	cell in vitro	10x 3' v1	McFaline-Figueroa_2019

**GSE117176**	10.1172/jci.insight.126453	21574	adipose tissue, bone marrow	10x 3' v2	Li_2019
**GSE117770**	10.1016/j.cmet.2019.01.021	30000	pancreas	10x 3' v2	Thompson_2019
**GSE120508**	10.1038/s41422-018-0099-2	6199	testis	10x 3' v2	Guo_2018
**GSE122342**	10.1016/j.stem.2018.12.015	11277	dorsal plus ventral thalamus	10x 3' v1	Xiang_2019
**GSE122960**	10.1164/rccm.201712-2410OC	80919	lung	10x 3' v2	Reyfman_2018
**GSE123722**	10.1016/j.cell.2020.11.017	9770	hindbrain	10x 3' v3	Andersen_2020
**GSE124691**	10.1016/j.celrep.2019.10.131	9804	lymphoid system	10x 3' v2	Magen_2019
**GSE128855**	10.1038/s41593-019-0393-4	21966	dura mater, brain, brain meninx, choroid plexus	10x 3' v2	Van Hove_2019
**GSE129519**	10.1038/s41586-019-1289-x	166242	telencephalon	10x 3' v2	Velasco_2019
**GSE130238**	10.1016/j.stem.2019.08.002	16086	cerebral cortex	10x 3' v2	Trujillo_2019
**GSE131685**	10.1038/s41597-019-0351-8	25404	kidney	10x 3' v2	Liao_2020
**GSE132672**	10.1038/s41586-020-1962-0	381350	prefrontal cortex, hippocampal formation, parietal cortex, occipital cortex, primary motor cortex, primary visual cortex, somatosensory cortex, frontal cortex, cerebral cortex, telencephalon, temporal cortex	10x 3' v2	Bhaduri_2020
**GSE135893**	10.1126/sciadv.aba1972	114396	lung parenchyma	10x 3' v2", "10x 5' v1	Habermann_2020
**GSE136001**	10.1038/s41467-021-21407-w	41059	brain	10x 3' v2", "10x 3' v3	Ochoka_2021
**GSE136103**	10.1038/s41586-019-1631-3	58358	liver	10x 3' v2	Ramachandran_2019
**GSE136831**	10.1126/sciadv.aba1983	312928	lung	10x 3' v2	Adams_2020
**GSE143317**	10.1038/s41591-021-01245-5	8020	bone marrow	10x 3' v2	Da Via_2021
**GSE145122**	10.1038/s41591-020-1043-9	45300	cerebral cortex	10x 3' v2	Khan_2020
**GSE147405**	10.1038/s41467-020-16066-2	97971	cell in vitro	10x 3' v2", "10x 3' v3	Cook_2020
**GSE149931**	10.1038/s41587-020-00763-w	27601	ventral part of telencephalon	10x 3' v3	Miura_2020

**GSE150903**	10.1126/science.aaz5626	32464	choroid plexus, telencephalon	10x 3' v3	Pellegrini_2020
**GSE156728**	10.1126/science.abe6474	183913	kidney, esophagus, skin epidermis, endometrium, ovary, fallopian tube, thyroid gland, blood, pancreas	10x 5' transcription profiling	Zheng_2022
**GSE156989**	10.1016/j.isci.2021.102404	487890	blood	10x 3' v3	Savage_2021
**GSE160641**	10.1126/sciadv.abg9518	10973	mammary gland	10x 3' v3	Opzoomer_2021
**GSE161125**	10.1038/s41467-020-20540-2	6904	bone marrow	10x 3' transcription profiling	Munoz-Rojas_2021
**GSE163973**	10.1038/s41467-021-24110-y	40655	dermis	10x 3' v3	Deng_2021
**GSE164690**	10.1038/s41467-021-27619-4	151680	neck	10x 3' v2	Kuerten_20221
**GSE165577**	10.1038/s41593-021-00906-5	58586	brain	10x 3' v3	Samarasinghe_2021
**GSE168323**	10.1038/s41467-021-27464-5	123294	midbrain tegmentum	10x 3' v3	Fiorenzano_2021
**GSE173351**	10.1038/s41586-021-03752-4	902463	blood, lung, brain, lymph node	10x 5' transcription profiling	Caushi_2021
**GSE187877**	10.1038/s41586-021-04330-4	6899	telencephalon	10x 3' v3	Kelava_2022
**GSE190604**	10.1126/science.abj4008	103805	blood	10x 3' v3	Schmidt_2022
**GSE201349**	10.1038/s41588-022-01088-x	826250	colon	10x 3' v3	Becker_2022
**GSE205554**	10.15252/embj.2022111118	12310	cerebral cortex	10x 3' v3	Vertesy_2022
	10.1126/science.adf1226	1665937	entorhinal cortex, head, midbrain, medulla oblongata, diencephalon, cortex, hypothalamus, telencephalon, forebrain, hippocampal formation, parietal cortex, occipital cortex,'dorsal plus ventral thalamus', cerebellum', striatum', 'pons', temporal cortex, frontal cortex, hindbrain, cerebral subcortex,	10x 3' v2", "10x 3' v3	Brau_2023

			telencephalon neural crest, brain, caudate-putamen		
	10.1016/j.devcel.2020.01.033	36807	lung	10x 3' v2	Miller_2020
	10.1038/s41586-022-05279-8	34088	brain	10x 3' v3	Fleck_2022
	10.1016/j.cell.2022.09.010	348379	cortical plate, brain telencephalon	10x 3' v2", "10x 3' v3	Uzquiano_2022
	10.1038/s41586-021-04358-6	663879	telencephalon	10x 3' v2", "10x 3' v3	Paulsen_2022
	10.1158/2159-8290.CD-21-0316	178630	liver	10x 3' v3	Wu_2022
	10.1186/s13059-020-02147-4	11512	brain	10x 3' v2	He_2020
	10.1016/j.cell.2020.08.001	102203	blood	10x 3' v3	Schulte-Schrepping_2020
	10.1371/journal.ppat.1008408	15085	blood	10x 3' v2	de Vries_2020
	10.1038/s41467-020-15543-y	43112	blood	10x 3' v2	Cano-Gamez_2020
	10.1038/s41592-021-01344-8	87051	brain	10x 3' v3	He_2022
	10.1038/s41586-019-1654-9	90576	brain	10x 3' v2	Kanton_2019
	10.1016/j.cell.2021.11.033	54975	lung	10x 3' v3	Wendisch_2021
	10.1038/s41587-021-01139-4	44429	brain	10x 3' v3	Kleshchevnikov_2020
	10.1016/j.medj.2022.05.002	102441	minor salivary gland, lung, synovial membrane of synovial joint, large intestine	10x 3' v2", "10x 3' v3	Korsunsky_2022

Suppl. Table 3 | Overview of the additional dissociated data extracted and harmonized from GEO and sfaira. Shown is the GEO ID used for downloading the data, the DOI, the number of cells before filtering low-quality cells, the primary tissue measured, the assay and the first author and publication year of the dataset and the respective publication.

Author/ Name	DOI / Link	Number of cells	Tissue	Assay	Species	N sections / N donors
Garrido-Trigo et al.	10.1038/s41467-023-40156-6	463,967	Bowel	CosMx	Human	171 / 9
Nanostring CosMx mouse brain	https://nanostring.com/products/cosmx-spatial-molecular-imager/ffpe-dataset/	86,916	Brain	CosMx	Mouse	130 / 2
Nanostring CosMx human liver	https://nanostring.com/products/cosmx-spatial-molecular-imager/ffpe-dataset/	793,318	Liver	CosMx	Human	2 / 2
Nanostring CosMx human lung	https://nanostring.com/products/cosmx-spatial-molecular-imager/ffpe-dataset/	771,236	Lung	CosMx	Human	8 / 5
Kukanja et al.	10.1016/j.cell.2024.02.030	592,421	Brain	ISS	Mouse	20 / 13
Ruiz-Moreno et al.	https://doi.org/10.1093/neuonc/noaf113	988,901	Brain	ISS	Human	13 / 13
Samakovlis et al.	10.21203/rs.3.rs-5046381/v1	260,398	Lung	ISS	Human	14 /14
Fang et al.	10.1126/science.abm1741	127,273	Brain	MERFISH	Human / Mouse	378 / 6
Allen et al.	10.1016/j.cell.2022.12.010	378,918	Brain	MERFISH	Mouse	783 / 12
Allen Institute brain atlas mouse p20	Unpublished	4,753,243	Brain	MERFISH	Mouse	59 / 1
Allen Institute brain atlas mouse p28	Unpublished	4,064,846	Brain	MERFISH	Mouse	55 / 1
Allen Institute brain atlas mouse female	Unpublished	4,925,098	Brain	MERFISH	Mouse	54 / 1
Vizgen MERFISH mouse brain	https://vizgen.com/data-release-program/	734,696	Brain	MERFISH	Mouse	9 / 3
Androvic et al.	10.1038/s41467-023-39447-9	347,968	Brain	MERFISH	Mouse	1646 / 3
Zhang et al.	10.1038/s41586-021-03705-x	276,556	Brain	MERFISH	Mouse	5000 / 2
Zhang et al.	10.1038/s41586-023-06808-9	9,343,457	Brain	MERFISH	Mouse	2151 / 4
Yao et al.	10.1038/s41586-023-06812-z	4,334,174	Brain	MERFISH	Mouse	59 / 1
Vizgen MERFISH human breast cancer	ttps://vizgen.com/data-release-program/	713.121	Breast	MERFISH	Human	1 / 1

Vizgen MERFISH human colon cancer	https://vizgen.com/data-release-program/	1,495,039	Colon	MERFISH	Human	2 / 2
Vizgen MERFISH human liver cancer	https://vizgen.com/data-release-program/	1,166,496	Liver	MERFISH	Human	2 / 2
Vizgen MERFISH human lung cancer	https://vizgen.com/data-release-program/	1,190,501	Lung	MERFISH	Human	2 / 2
Vizgen MERFISH human ovarian cancer	https://vizgen.com/data-release-program/	896,638	Ovary	MERFISH	Human	4 / 2
Vizgen MERFISH human prostate cancer	https://vizgen.com/data-release-program/	1,715,493	Prostate	MERFISH	Human	2 / 2
Vizgen MERFISH human skin cancer	https://vizgen.com/data-release-program/	676,007	Skin	MERFISH	Human	2 / 2
Vizgen MERFISH human uterine cancer	https://vizgen.com/data-release-program/	2,343,540	Uterus	MERFISH	Human	3 / 2
Choi et al.	10.1038/s41467-023-40674-3	367,369	Retina	MERFISH	Mouse	17 / 12
Magen et al.	10.1038/s41591-023-02345-0	1.671.375	Liver	MERFISH	Human	10 / 7
10x Genomics Xenium human brain	https://www.10xgenomics.com/datasets	110248	Brain	Xenium	Human	3 / 3
10x Genomics Xenium mouse brain - Alzheimers	https://www.10xgenomics.com/datasets	351.714	Brain	Xenium	Mouse	6 / 6
10x Genomics Xenium mouse brain	https://www.10xgenomics.com/datasets	474.734	Brain	Xenium	Mouse	3 / 1
10x Genomics Xenium breast cancer (add-on)	https://www.10xgenomics.com/datasets	2,721,056	Breast	Xenium	Human	4 / 3
10x Genomics Xenium colon cancer (add-on)	https://www.10xgenomics.com/datasets	1,234,639	Colon	Xenium	Human	2 / 2
10x Genomics Xenium healthy colon (add-on)	https://www.10xgenomics.com/datasets	546,806	Colon	Xenium	Human	2 / 2
10x Genomics Xenium kidney cancer (add-on)	https://www.10xgenomics.com/datasets	56.510	Kidney	Xenium	Human	1 / 1
10x Genomics Xenium healthy	https://www.10xgenomics.com/datasets	97.560	Kidney	Xenium	Human	1 / 1

kidney (add-on)						
10x Genomics Xenium lung cancer (add-on)	https://www.10xgenomics.com/datasets	681,530	Lung	Xenium	Human	2 / 2
10x Genomics Xenium healthy lung (add-on)	https://www.10xgenomics.com/datasets	295.883	Lung	Xenium	Human	1 / 1
10x Genomics Xenium lymph node	https://www.10xgenomics.com/datasets	377.985	Lymph Node	Xenium	Human	1 / 1
10x Genomics Xenium pancreas cancer	https://www.10xgenomics.com/datasets	190.965	Pancreas	Xenium	Human	1 / 1
10x Genomics Xenium healthy pancreas	https://www.10xgenomics.com/datasets	103901	Pancreas	Xenium	Human	1 / 1
10x Genomics Xenium skin cancer (add-on)	https://www.10xgenomics.com/datasets	194,479	Skin	Xenium	Human	2 / 2
Marco Salas et al.	https://doi.org/10.5281/zenodo.10566172	624,058	Brain	Xenium	Mouse	1 / 1
Kukanja et al.	10.1016/j.cell.2024.02.030	660,801	Spinal Cord	Xenium	Human	6 / 6

Suppl. Table 4 | Overview of the spatial datasets extracted and harmonized. Shown is the author name of the original publication or the name describing the dataset, the DOI or dataset retrieval link used for downloading the data, the number of cells, tissue, assay, species, and the number of tissue sections and donors/ animals measured in the dataset. For the overview datasets obtained via the Vizgen data release and the 10x Genomics data resource are grouped by tissue and condition to reduce table size. Datasets highlighted in blue mark the dataset used for downstream Nicheformer tasks.

Hyperparameter	Nicheformer (fine-tuned) & Nicheformer (linear probing)	Nicheformer (MLP)	scVI (linear probing)	PCA (linear probing)
Optimizer	AdamW	Adam	Adam	Adam
Optimizer momentum	$\beta_1 = 0.9$<br>$\beta_2 = 0.99$	$\beta_1 = 0.9$<br>$\beta_2 = 0.99$	$\beta_1 = 0.9$<br>$\beta_2 = 0.99$	$\beta_1 = 0.9$<br>$\beta_2 = 0.99$
Maximum learning rate	1e-4	1e-3	1e-3	1e-3
Minimum learning rate	1e-5	1e-3	1e-3	1e-3
Cosine decay length	1 epoch	-	-	-
Weight decay	0.1	0001	0.001	0.001
Dropout	0.0	-	-	-
Maximum gradient clipping	1.0	-	-	-
Batch size	9	256	256	256
Gradient accumulation	10	-	-	-
Hidden dimensions	-	256	-	-
Precision	bfloat16	16-mixed	16-mixed	16-mixed
Epochs	1	5	1	1

Suppl. Table 5 | Overview of the fine-tuning and linear probing hyperparameters. Shown are the model hyperparameters used for the different Nicheformer downstream tasks and the model parameters used for MLP and linear probing models trained on scVI and PCA embeddings.
