# Supplementary material for: Nicheformer: a foundation model for single-cell and spatial omics
Source: Nat Methods. 2025 Oct 30;22(12):2525–38. doi: 10.1038/s41592-025-02814-z (PMC12695652; doi:10.1038/s41592-025-02814-z)
Supplement: Supplementary file 2 — Reporting Summary [file 41592_2025_2814_MOESM2_ESM.pdf]

nature portfolio

Corresponding author(s): Fabian Theis

Last updated by author(s): 5/5/2025

Reporting Summary

Nature Portfolio wishes to improve the reproducibility of the work that we publish. This form provides structure for consistency and transparency in reporting. For further information on Nature Portfolio policies, see our Editorial Policies and the Editorial Policy Checklist.

Statistics

For all statistical analyses, confirm that the following items are present in the figure legend, table legend, main text, or Methods section.

n/a	Confirmed	
☐	☒	The exact sample size (*n*) for each experimental group/condition, given as a discrete number and unit of measurement
☐	☒	A statement on whether measurements were taken from distinct samples or whether the same sample was measured repeatedly
☐	☒	The statistical test(s) used AND whether they are one- or two-sided<br>*Only common tests should be described solely by name; describe more complex techniques in the Methods section.*
☐	☒	A description of all covariates tested
☐	☒	A description of any assumptions or corrections, such as tests of normality and adjustment for multiple comparisons
☐	☒	A full description of the statistical parameters including central tendency (e.g. means) or other basic estimates (e.g. regression coefficient) AND variation (e.g. standard deviation) or associated estimates of uncertainty (e.g. confidence intervals)
☐	☒	For null hypothesis testing, the test statistic (e.g. *F*, *t*, *r*) with confidence intervals, effect sizes, degrees of freedom and *P* value noted<br>*Give P values as exact values whenever suitable.*
☒	☐	For Bayesian analysis, information on the choice of priors and Markov chain Monte Carlo settings
☒	☐	For hierarchical and complex designs, identification of the appropriate level for tests and full reporting of outcomes
☒	☐	Estimates of effect sizes (e.g. Cohen's *d*, Pearson's *r*), indicating how they were calculated

Our web collection on statistics for biologists contains articles on many of the points above.

Software and code

Policy information about availability of computer code

Data collection	*Provide a description of all commercial, open source and custom code used to collect the data in this study, specifying the version used OR state that no software was used.*
Data analysis	Python 3.10, Pytorch 2.3, scikit-learn 1.6.1, scGPT 0.2.1, GeneFormer official HF, scvi-tools 1.2.0

For manuscripts utilizing custom algorithms or software that are central to the research but not yet described in published literature, software must be made available to editors and reviewers. We strongly encourage code deposition in a community repository (e.g. GitHub). See the Nature Portfolio guidelines for submitting code & software for further information.

Data

Policy information about availability of data

All manuscripts must include a data availability statement. This statement should provide the following information, where applicable:

- Accession codes, unique identifiers, or web links for publicly available datasets
- A description of any restrictions on data availability
- For clinical datasets or third party data, please ensure that the statement adheres to our policy

The Allen brain atlas consortium generated the Allen Institute brain atlas mouse p20, Allen Institute brain atlas mouse p28, and Allen Institute brain atlas mouse female datasets (Suppl. Table 4), which have kindly been provided to us prior to publication. The Xenium human spinal cord, the ISS human brain GBM, the ISS human discover healthy lung, the ISS mouse EAE MS, and the Xenium mouse brain datasets have been generated by Mat Nillson lab and have been kindly provided

to us prior to publication.
All datasets used in this study are publicly available. The single-cell RNA sequencing data can be accessed through the Gene Expression Omnibus (GEO) under the following accession numbers: GSE117824 (DOI: 10.1038/s41586-019-1367-0), GSE118068 (DOI: 10.1038/s41586-019-1158-7), GSE119940 (DOI: 10.1038/s41590-019-0403-4), GSE124952 (DOI: 10.1038/s41467-019-12054-3), GSE126060 (DOI: 10.1038/s41467-019-14172-4), GSE128423 (DOI: 10.1016/j.cell.2019.04.040), GSE128761 (DOI: 10.1016/j.stem.2020.08.001), GSE128987 (DOI: 10.1038/s41556-020-00619-0), GSE129826 (DOI: 10.1016/j.celrep.2019.10.073), GSE130593 (DOI: 10.1242/dev.183251), GSE130822 (DOI: 10.1016/j.stem.2019.12.011), GSE130879 (DOI: 10.1016/j.immuni.2019.12.002), GSE130888 (DOI: 10.1126/scitranslmed.aav5341), GSE131339 (DOI: 10.1038/s41467-019-13465-y), GSE131996 (DOI: 10.1016/j.immuni.2019.06.009), GSE132355 (DOI: 10.1038/s41467-020-18231-z), GSE133531 (DOI: 10.1038/s41588-019-0531-7), GSE134571 (DOI: 10.1038/s41586-019-1535-2), GSE135310 (DOI: 10.1161/CIRCRESAHA.120.317200), GSE135326 (DOI: 10.1038/s41586-019-1644-y), GSE135356 (DOI: 10.1038/s41467-020-18957-w), GSE135414 (DOI: 10.1016/j.celrep.2020.01.075), GSE136394 (DOI: 10.1158/2326-6066.CIR19-0299), GSE136441 (DOI: 10.1073/pnas.2005570117), GSE137026 (DOI: 10.1084/jem.20220126), GSE139168 (DOI: 10.1126/sciadv.aba9950), GSE140510 (DOI: 10.1038/s41591-019-0695-9), GSE140628 (DOI: 10.1158/2159-8290.CD19-0958), GSE141471 (DOI: 10.1038/s41467-021-27899-w), GSE141526 (DOI: 10.1126/sciadv.abm7981), GSE141552 (DOI: 10.1093/hmg/ddaa038), GSE141784 (DOI: 10.1084/jem.20192362), GSE142143 (DOI: 10.1038/s41419-022-04693-0), GSE142797 (DOI: 10.1101/2020.04.27.063503), GSE143293 (DOI: 10.1038/s41586-020-3017-y), GSE145216 (DOI: 10.1016/j.cell.2020.03.004), GSE145251 (DOI: 10.1016/j.stem.2022.03.001), GSE145326 (DOI: 10.1172/JCI130323), GSE145689 (DOI: 10.1681/ASN.2020070930), GSE145866 (DOI: 10.1016/j.celrep.2020.107952), GSE146122 (DOI: 10.1016/j.cell.2020.03.015), GSE146138 (DOI: 10.1053/j.gastro.2020.09.011), GSE146194 (DOI: 10.1016/j.cell.2022.05.013), GSE146298 (DOI: 10.1016/j.celrep.2020.03.059), GSE146512 (DOI: 10.1016/j.celrep.2020.108027), GSE148339 (DOI: 10.1016/j.jcmgh.2020.07.012), GSE148978 (DOI: 10.1016/j.immuni.2020.10.024), GSE149040 (DOI: 10.1038/s41467-021-21704-4), GSE149201 (DOI: 10.1158/2159-8290.CD-20-0461), GSE149356 (DOI: 10.1126/sciimmunol.abf0125), GSE149931 (DOI: 10.1038/s41587-020-00763-w), GSE150708 (DOI: 10.21203/rs.3.rs-62758/v1), GSE150871 (DOI: 10.1038/s41586-020-2795-6), GSE150995 (DOI: 10.1172/JCI136142), GSE151186 (DOI: 10.1016/j.stem.2020.10.003), GSE152325 (DOI: 10.1038/s41556-020-00617-2), GSE152573 (DOI: 10.1182/blood.2020007747), GSE152988 (DOI: 10.1038/s41593-021-00862-0), GSE152999 (DOI: 10.1038/s41467-020-20351-5), GSE153099 (DOI: 10.1016/j.stemcr.2020.12.018), GSE153117 (DOI: 10.1038/s41467-021-26069-2), GSE153274 (DOI: 10.1038/s41467-021-22021-6), GSE153288 (DOI: 10.1038/s41467-020-17544-3), GSE153762 (DOI: 10.7554/eLife.60223), GSE153770 (DOI: 10.1016/j.celrep.2020.108004), GSE153802, GSE154196 (DOI: 10.15252/embj.2020106423), GSE154359 (DOI: 10.1016/j.stem.2020.08.015), GSE154386 (DOI: 10.1371/journal.ppat.1009240), GSE154567 (DOI: 10.1016/j.celrep.2020.108590), GSE154579 (DOI: 10.1038/s41467-020-19234-6), GSE154932 (DOI: 10.1088/1478-3975/abb09c), GSE155226 (DOI: 10.1126/scitranslmed.abf7872), GSE155340 (DOI: 10.1126/sciimmunol.abb5168), GSE155788 (DOI: 10.7554/eLife.61413), GSE155850 (DOI: 10.1172/jci.insight.139932), GSE156136 (DOI: 10.1016/j.cell.2020.09.062), GSE156183 (DOI: 10.1073/pnas.2017742118), GSE156245 (DOI: 10.1038/s41586-021-03283-y), GSE156285 (DOI: 10.1126/sciimmunol.abc6259), GSE156920 (DOI: 10.1038/s41467-021-23320-8), GSE157244 (DOI: 10.1161/JAHA.120.019019), GSE157292 (DOI: 10.1172/jci.insight.141321), GSE157362 (DOI: 10.1242/dev.197111), GSE157525 (DOI: 10.1101/gad.339978.120), GSE157771 (DOI: 10.1038/s41593-020-00745-w), GSE157773, GSE157977 (DOI: 10.1126/science.aaz6063), GSE158038 (DOI: 10.1038/s41467-021-22210-3), GSE158192 (DOI: 10.1038/s41467-021-22817-6), GSE158356_mouse (DOI: 10.26508/lsa.202000935), GSE158450 (DOI: 10.1038/s41467-020-20343-5), GSE159354 (DOI: 10.1016/j.xcrm.2020.100140), GSE159519 (DOI: 10.1016/j.cell.2020.10.030), GSE159977 (DOI: 10.1038/s41586-021-03362-0), GSE160061 (DOI: 10.1111/cpr.12933), GSE160097 (DOI: 10.1002/eji.202048797), GSE160098 (DOI: 10.1038/s41467-023-38647-7), GSE160664 (DOI: 10.1164/rccm.202008-31980C), GSE160729 (DOI: 10.1016/j.cmet.2020.12.004), GSE160772 (DOI: 10.1096/fj.202002123R), GSE161066 (DOI: 10.3389/fphys.2021.637924), GSE161227 (DOI: 10.1084/jem.20212479), GSE161230, GSE161363 (DOI: 10.1126/science.abc1944), GSE161685 (DOI: 10.1172/jci.insight.144294), GSE161937 (DOI: 10.1073/pnas.1915389116), GSE162073 (DOI: 10.1084/jem.20200844), GSE162807 (DOI: 10.1038/s41467-022-28473-8), GSE163018 (DOI: 10.1038/s41421-021-00266-1), GSE163278 (DOI: 10.1172/jci.insight.127807), GSE163650 (DOI: 10.1371/journal.pone.0244743), GSE163668 (DOI: 10.1038/s41586-021-03234-7), GSE163701 (DOI: 10.1038/s41698-021-00160-9), GSE163830, GSE163919, GSE164044 (DOI: 10.1016/j.neuron.2019.08.002), GSE164573 (DOI: 10.1038/s41467-021-22842-5), GSE165551 (DOI: 10.7554/eLife.67436), GSE165554 (DOI: 10.7554/eLife.67436), GSE166218 (DOI: 10.1093/neuonc/noac138), GSE166262 (DOI: 10.1038/s41588-021-00818-x), GSE166525 (DOI: 10.1186/s13046-023-02686-1), GSE166797 (DOI: 10.1073/pnas.2023070118), GSE166992 (DOI: 10.1016/j.celrep.2021.108863), GSE167595 (DOI: 10.1158/1940-6207.CAPR-21-0378), GSE167992 (DOI: 10.1016/j.stem.2021.04.003), GSE168732 (DOI: 10.1038/s41467-021-25771-5), GSE168758 (DOI: 10.1016/j.jhep.2021.03.029), GSE169718 (DOI: 10.1016/j.devcel.2021.12.012), GSE172127 (DOI: 10.1038/s41421-021-00266-1), GSE200218 (DOI: 10.1016/j.cell.2022.06.007), GSE225278 (DOI: 10.1038/s41467-023-38704-1), GSE114687 (DOI: 10.1038/s41588-019-0489-5), GSE117176 (DOI: 10.1172/jci.insight.126453), GSE117770 (DOI: 10.1016/j.cmet.2019.01.021), GSE120508 (DOI: 10.1038/s41422-018-0099-2), GSE122342 (DOI: 10.1016/j.stem.2018.12.015), GSE122960 (DOI: 10.1164/rccm.201712-24100C), GSE123722 (DOI: 10.1016/j.cell.2020.11.017), GSE124691 (DOI: 10.1016/j.celrep.2019.10.131), GSE128855 (DOI: 10.1038/s41593-019-0393-4), GSE129519 (DOI: 10.1038/s41586-019-1289-x), GSE130238 (DOI: 10.1016/j.stem.2019.08.002), GSE131685 (DOI: 10.1038/s41597-019-0351-8), GSE132672 (DOI: 10.1038/s41586-020-1962-0), GSE135893 (DOI: 10.1126/sciadv.aba1972), GSE136001 (DOI: 10.1038/s41467-021-21407-W), and GSE136103 (DOI: 10.1038/s41586-019-1631-3).
The Allen brain atlas consortium generated the Allen Institute brain atlas mouse p20, Allen Institute brain atlas mouse p28, and Allen Institute brain atlas mouse female datasets (Suppl. Table 4), which have kindly been provided to us prior to publication. The Xenium human spinal cord, the ISS human brain GBM, the ISS human discover healthy lung, the ISS mouse EAE MS, and the Xenium mouse brain datasets have been generated by Mat Nillson lab and have been kindly provided to us prior to publication.
All datasets used in this study are publicly available. The single-cell RNA sequencing data can be accessed through the Gene Expression Omnibus (GEO) under the following accession numbers: GSE117824 (DOI: 10.1038/s41586-019-1367-0), GSE118068 (DOI: 10.1038/s41586-019-1158-7), GSE119940 (DOI: 10.1038/s41590-019-0403-4), GSE124952 (DOI: 10.1038/s41467-019-12054-3), GSE126060 (DOI: 10.1038/s41467-019-14172-4), GSE128423 (DOI: 10.1016/j.cell.2019.04.040), GSE128761 (DOI: 10.1016/j.stem.2020.08.001), GSE128987 (DOI: 10.1038/s41556-020-00619-0), GSE129826 (DOI: 10.1016/j.celrep.2019.10.073), GSE130593 (DOI: 10.1242/dev.183251), GSE130822 (DOI: 10.1016/j.stem.2019.12.011), GSE130879 (DOI: 10.1016/j.immuni.2019.12.002), GSE130888 (DOI: 10.1126/scitranslmed.aav5341), GSE131339 (DOI: 10.1038/s41467-019-13465-y), GSE131996 (DOI: 10.1016/j.immuni.2019.06.009), GSE132355 (DOI: 10.1038/s41467-020-18231-z), GSE133531 (DOI: 10.1038/s41588-019-0531-7), GSE134571 (DOI: 10.1038/s41586-019-1535-2), GSE135310 (DOI: 10.1161/CIRCRESAHA.120.317200), GSE135326 (DOI: 10.1038/s41586-019-1644-y), GSE135356 (DOI: 10.1038/s41467-020-18957-w), GSE135414 (DOI: 10.1016/j.celrep.2020.01.075), GSE136394 (DOI: 10.1158/2326-6066.CIR19-0299), GSE136441 (DOI: 10.1073/pnas.2005570117), GSE137026 (DOI: 10.1084/jem.20220126), GSE139168 (DOI: 10.1126/sciadv.aba9950), GSE140510 (DOI: 10.1038/s41591-019-0695-9), GSE140628 (DOI: 10.1158/2159-8290.CD19-0958), GSE141471 (DOI: 10.1038/s41467-021-27899-w), GSE141526 (DOI: 10.1126/sciadv.abm7981), GSE141552 (DOI: 10.1093/hmg/ddaa038), GSE141784 (DOI: 10.1084/jem.20192362), GSE142143 (DOI: 10.1038/s41419-022-04693-0), GSE142797 (DOI: 10.1101/2020.04.27.063503), GSE143293 (DOI: 10.1038/s41586-020-3017-y), GSE145216 (DOI: 10.1016/j.cell.2020.03.004), GSE145251 (DOI: 10.1016/j.stem.2022.03.001), GSE145326 (DOI: 10.1172/JCI130323), GSE145689 (DOI: 10.1681/ASN.2020070930), GSE145866 (DOI: 10.1016/j.celrep.2020.107952), GSE146122 (DOI: 10.1016/j.cell.2020.03.015), GSE146138 (DOI: 10.1053/j.gastro.2020.09.011), GSE146194 (DOI: 10.1016/j.cell.2022.05.013), GSE146298 (DOI: 10.1016/j.celrep.2020.03.059), GSE146512 (DOI: 10.1016/j.celrep.2020.108027), GSE148339 (DOI: 10.1016/j.jcmgh.2020.07.012), GSE148978 (DOI: 10.1016/j.immuni.2020.10.024), GSE149040 (DOI: 10.1038/s41467-021-21704-4), GSE149201 (DOI: 10.1158/2159-8290.CD-20-0461), GSE149356 (DOI: 10.1126/sciimmunol.abf0125), GSE149931 (DOI: 10.1038/s41587-020-00763-w), GSE150708 (DOI: 10.21203/rs.3.rs-62758/v1), GSE150871 (DOI: 10.1038/s41586-020-2795-6), GSE150995 (DOI: 10.1172/JCI136142), GSE151186 (DOI: 10.1016/j.stem.2020.10.003), GSE152325 (DOI: 10.1038/s41556-020-00617-2), GSE152573 (DOI: 10.1182/blood.2020007747), GSE152988 (DOI: 10.1038/s41593-021-00862-0), GSE152999 (DOI: 10.1038/s41467-020-20351-5), GSE153099 (DOI: 10.1016/j.stemcr.2020.12.018), GSE153117 (DOI: 10.1038/s41467-021-26069-2), GSE153274 (DOI: 10.1038/s41467-021-22021-6), GSE153288 (DOI: 10.1038/s41467-020-17544-3), GSE153762 (DOI: 10.7554/eLife.60223), GSE153770 (DOI: 10.1016/j.celrep.2020.108004), GSE153802, GSE154196 (DOI: 10.15252/embj.2020106423), GSE154359 (DOI: 10.1016/j.stem.2020.08.015), GSE154386 (DOI: 10.1371/journal.ppat.1009240), GSE154567 (DOI: 10.1016/j.celrep.2020.108590), GSE154579 (DOI: 10.1038/s41467-020-19234-6), GSE154932 (DOI: 10.1088/1478-3975/abb09c), GSE155226 (DOI: 10.1126/scitranslmed.abf7872), GSE155340 (DOI: 10.1126/sciimmunol.abb5168), GSE155788 (DOI: 10.7554/eLife.61413), GSE155850 (DOI: 10.1172/jci.insight.139932), GSE156136 (DOI: 10.1016/j.cell.2020.09.062), GSE156183 (DOI: 10.1073/pnas.2017742118), GSE156245 (DOI: 10.1038/s41586-021-03283-y),

GSE156285 (DOI: 10.1126/sciimmunol.abc6259), GSE156920 (DOI: 10.1038/s41467-021-23320-8), GSE157244 (DOI: 10.1161/JAHA.120.019019), GSE157292 (DOI: 10.1172/jci.insight.141321), GSE157362 (DOI: 10.1242/dev.197111), GSE157525 (DOI: 10.1101/gad.339978.120), GSE157771 (DOI: 10.1038/s41593-020-00745-w), GSE157773, GSE157977 (DOI: 10.1126/science.aaz6063), GSE158038 (DOI: 10.1038/s41467-021-22210-3), GSE158192 (DOI: 10.1038/s41467-021-22817-6), GSE158356_mouse (DOI: 10.26508/lsa.202000935), GSE158450 (DOI: 10.1038/s41467-020-20343-5), GSE159354 (DOI: 10.1016/j.xcrm.2020.100140), GSE159519 (DOI: 10.1016/j.cell.2020.10.030), GSE159977 (DOI: 10.1038/s41586-021-03362-0), GSE160061 (DOI: 10.1111/cpr.12933), GSE160097 (DOI: 10.1002/eji.202048797), GSE160098 (DOI: 10.1038/s41467-023-38647-7), GSE160664 (DOI: 10.1164/rccm.202008-31980C), GSE160729 (DOI: 10.1016/j.cmet.2020.12.004), GSE160772 (DOI: 10.1096/fj.202002123R), GSE161066 (DOI: 10.3389/fphys.2021.637924), GSE161227 (DOI: 10.1084/jem.20212479), GSE161230, GSE161363 (DOI: 10.1126/science.abc1944), GSE161685 (DOI: 10.1172/jci.insight.144294), GSE161937 (DOI: 10.1073/pnas.1915389116), GSE162073 (DOI: 10.1084/jem.20200844), GSE162807 (DOI: 10.1038/s41467-022-28473-8), GSE163018 (DOI: 10.1038/s41421-021-00266-1), GSE163278 (DOI: 10.1172/jci.insight.127807), GSE163650 (DOI: 10.1371/journal.pone.0244743), GSE163668 (DOI: 10.1038/s41586-021-03234-7), GSE163701 (DOI: 10.1038/s41698-021-00160-9), GSE163830, GSE163919, GSE164044 (DOI: 10.1016/j.neuron.2019.08.002), GSE164573 (DOI: 10.1038/s41467-021-22842-5), GSE165551 (DOI: 10.7554/eLife.67436), GSE165554 (DOI: 10.7554/eLife.67436), GSE166218 (DOI: 10.1093/neuonc/noac138), GSE166262 (DOI: 10.1038/s41588-021-00818-x), GSE166525 (DOI: 10.1186/s13046-023-02686-1), GSE166797 (DOI: 10.1073/pnas.2023070118), GSE166992 (DOI: 10.1016/j.celrep.2021.108863), GSE167595 (DOI: 10.1158/1940-6207.CAPR-21-0378), GSE167992 (DOI: 10.1016/j.stem.2021.04.003), GSE168732 (DOI: 10.1038/s41467-021-25771-5), GSE168758 (DOI: 10.1016/j.jhep.2021.03.029), GSE169718 (DOI: 10.1016/j.devcel.2021.12.012), GSE172127 (DOI: 10.1038/s41421-021-00266-1), GSE200218 (DOI: 10.1016/j.cell.2022.06.007), GSE225278 (DOI: 10.1038/s41467-023-38704-1), GSE114687 (DOI: 10.1038/s41588-019-0489-5), GSE117176 (DOI: 10.1172/jci.insight.126453), GSE117770 (DOI: 10.1016/j.cmet.2019.01.021), GSE120508 (DOI: 10.1038/s41422-018-0099-2), GSE122342 (DOI: 10.1016/j.stem.2018.12.015), GSE122960 (DOI: 10.1164/rccm.201712-24100C), GSE123722 (DOI: 10.1016/j.cell.2020.11.017), GSE124691 (DOI: 10.1016/j.celrep.2019.10.131), GSE128855 (DOI: 10.1038/s41593-019-0393-4), GSE129519 (DOI: 10.1038/s41586-019-1289-x), GSE130238 (DOI: 10.1016/j.stem.2019.08.002), GSE131685 (DOI: 10.1038/s41597-019-0351-8), GSE132672 (DOI: 10.1038/s41586-020-1962-0), GSE135893 (DOI: 10.1126/sciadv.aba1972), GSE136001 (DOI: 10.1038/s41467-021-21407-W), and GSE136103 (DOI: 10.1038/s41586-019-1631-3).

Human research participants

Policy information about studies involving human research participants and Sex and Gender in Research.

Reporting on sex and gender	N/A
Population characteristics	N/A
Recruitment	N/A
Ethics oversight	N/A

Note that full information on the approval of the study protocol must also be provided in the manuscript.

Field-specific reporting

Please select the one below that is the best fit for your research. If you are not sure, read the appropriate sections before making your selection.

☒ Life sciences ☐ Behavioural & social sciences ☐ Ecological, evolutionary & environmental sciences

For a reference copy of the document with all sections, see nature.com/documents/nr-reporting-summary-flat.pdf

Life sciences study design

All studies must disclose on these points even when the disclosure is negative.

Sample size	No power analysis was performed to select sample size. However, we followed standard computational practices using at least 3 random seeds for comparing models. For the statistical analysis performed in the attention analysis section, we sampled at least 2,000 cells from each tissue and condition to have a sufficiently large sample size of attention scores to obtain robust results. For the rest of spatial predictive tasks (e.g. niche composition prediction), entire spatial slices were held-out and the performance of the model evaluated in all the cells of those spatial slices. For the label transfer tasks, we employed a dissociated data with more than 7,000 cells to ensure a sufficient sample size.
Data exclusions	No data was excluded from analyses.
Replication	Replication of the results were done, running different random seeds for each analysis, including splits. Furthermore, since both data, model weights and code are provided, all results can be reproduced using the official github repo.
Randomization	Randomization was done through random seeding. Also, covariates were controlled to ensure no data leakage. For instance, in the case of the spatial tasks (e.g. niche classification), it was controlled that no test data was leakage into training and validation sets. Furthermore, the splitting was done at random, sampling spatial slices randomly. Likewise for the attention analyses, all cells were randomly sampled.
Blinding	Blinding not relevant in the study. Train, validation, test splits were done at random. For attention analyses, all cells were also randomly sampled.

Reporting for specific materials, systems and methods

We require information from authors about some types of materials, experimental systems and methods used in many studies. Here, indicate whether each material, system or method listed is relevant to your study. If you are not sure if a list item applies to your research, read the appropriate section before selecting a response.

Materials & experimental systems

n/a	Involved in the study
☒	☐ Antibodies
☒	☐ Eukaryotic cell lines
☒	☐ Palaeontology and archaeology
☒	☐ Animals and other organisms
☒	☐ Clinical data
☒	☐ Dual use research of concern

Methods

n/a	Involved in the study
☒	☐ ChIP-seq
☒	☐ Flow cytometry
☒	☐ MRI-based neuroimaging
